# Unveiling roles of beneficial gut bacteria and optimal diets for health

**DOI:** 10.3389/fmicb.2025.1527755

**Published:** 2025-02-18

**Authors:** Suresh Kumar, Riya Mukherjee, Pratibha Gaur, Élcio Leal, Xiaoming Lyu, Saheem Ahmad, Paridhi Puri, Chung-Ming Chang, V. Samuel Raj, Ramendra Pati Pandey

**Affiliations:** ^1^National Institute of Biologicals, Ministry of Health & Family Welfare, Govt. of India, Noida, India; ^2^Graduate Institute of Biomedical Sciences, Chang Gung University, Taoyuan, Taiwan; ^3^Department of Medical Biotechnology and Laboratory Science, Chang Gung University, Taoyuan, Taiwan; ^4^Centre for Drug Design Discovery and Development (C4D), SRM University Delhi-NCR, Sonepat, India; ^5^Department of Biotechnology and Microbiology, SRM University Delhi-NCR, Sonepat, India; ^6^Laboratório de Diversidade Viral, Instituto de Ciências Biológicas, Universidade Federal Do Pará, Belém, Brazil; ^7^Department of Laboratory Medicine, The Third Affiliated Hospital, Southern Medical University, Guangzhou, China; ^8^Department of Medical Laboratory Sciences, College of Applied Medical Sciences, University of Hail, Ha'il, Saudi Arabia; ^9^University Centre for Research and Development, Chandigarh University, Mohali, India; ^10^Master & Ph.D Program in Biotechnology Industry, Chang Gung University, Taoyuan, Taiwan

**Keywords:** healthy gut bacteria, phytochemicals, prebiotic foods and probiotic foods, probiotics, fatty acid

## Abstract

The gut microbiome plays a pivotal role in human health, influencing digestion, immunity, and disease prevention. Beneficial gut bacteria such as *Akkermansia muciniphila*, *Adlercreutzia equolifaciens*, and *Christensenella minuta* contribute to metabolic regulation and immune support through bioactive metabolites like short-chain fatty acids (SCFAs). Dietary patterns rich in prebiotics, fermented foods, and plant-based bioactive compounds, including polyphenols and flavonoids, promote microbiome diversity and stability. However, challenges such as individual variability, bioavailability, dietary adherence, and the dynamic nature of the gut microbiota remain significant. This review synthesizes current insights into gut bacteria’s role in health, emphasizing the mechanisms by which dietary interventions modulate microbiota. Additionally, it highlights advancements in microbiome-targeted therapies and the transformative potential of personalized nutrition, leveraging microbiota profiling and artificial intelligence (AI) to develop tailored dietary strategies for optimizing gut health and mitigating chronic inflammatory disorders. Addressing these challenges requires a multidisciplinary approach that integrates scientific innovation, ethical frameworks, and practical implementation strategies.

## 1 Introduction

**Figure 1:**
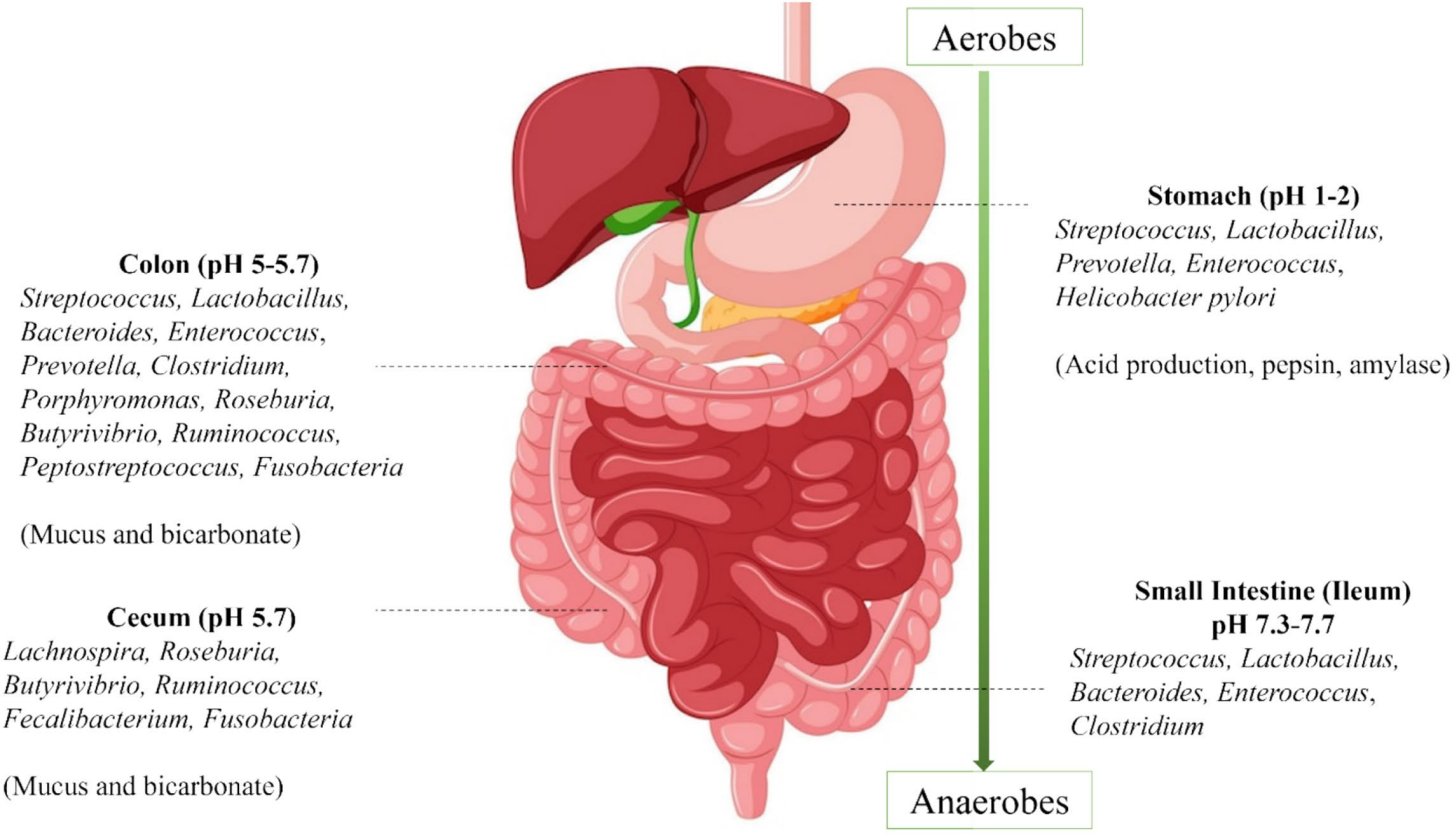
Distribution of gut microbiota.

The human gut is a fascinating ecosystem that harbors trillions of microorganisms collectively known as gut microbiota that play a crucial role in several physiological functions through their derived metabolites such as nutrient metabolism, immune system regulation, vitamin production, mental health, and brain function that contribute to overall maintaining of health. There is a wide distribution of microbes in the gut which is essential for better health (Figure 1). Some of the key gut bacteria, including *Akkermansia muciniphila, Adlercreutzia equolifaciens, Barnesiella, Christensenella minuta, and Oxalobacter formigenes*, *Lactobacillus, Bifidobacterium, Faecalibacterium prausnitzii, Roseburia* spp. contribute to unusual health benefits such as improved gut barrier function, better glucose metabolism, reduced inflammation, weight management, and prevention of kidney stone formation (Anhê et al., 2016; Maruo et al., 2008; Ubeda et al., 2013; Ang et al., 2023; Kaufman et al., 2008).

The dietary sources such as polyphenols, alkaloids, capsaicin, and polysaccharides are essential constituents that support the growth of these beneficial bacteria, leading to improved digestive health and overall well-being (Chenbo et al., 2022; Othman et al., 2019). The metabolites are small molecules that produced by the gut microbiota as byproducts can target directly as well as indirectly both the bacteria themselves and the host, regulating the composition and function of the gut microbiota (Liu et al., 2022; Jandhyala et al., 2015). In recent years, the significance of gut microbiota and their metabolites in influencing human health has garnered significant attention.

The role of gut microbiota in disease initiation is gaining significant attention. Dysbiosis, an imbalance in microbial composition, is strongly associated with the onset of conditions such as inflammatory bowel disease (IBD), metabolic syndrome, and neurodegenerative disorders. Altered microbial populations lead to reduced production of beneficial metabolites like SCFAs and increased levels of pro-inflammatory compounds such as lipopolysaccharides (LPS), disrupting gut barrier integrity and systemic homeostasis (Tan et al., 2014). Additionally, microbial interactions with dietary components such as fibers, polyphenols, and omega-3 fatty acids further influence health outcomes by modulating inflammation, immune signaling, and metabolic pathways (Sultan et al., 2021).

Several studies have focused on elucidating the specific functions and mechanisms of these microorganisms in relation to various diseases (Lavelle and Sokol, 2020; Parada Venegas et al., 2019; Liu et al., 2022). There is currently a lack of a comprehensive summarization regarding the most beneficial gut bacteria, their derived metabolites, and their dietary sources that contribute to maintaining overall health. In this review, we provide the potential role of microbiota in human health. We also discuss the essential gut bacteria and emphasize the derived metabolites of these gut bacteria especially the functions of regulating the local and systemic immune system, energy metabolism, and neural activity. Finally, we discuss how dietary approaches impacts the gut microbial ecology, and foods that promote healthier and more resilient intestinal gut bacteria.

## 2 Impact of microbiota on human health

The microbial balance in the intestine is closely related to human diseases and wellness. Extensive research has uncovered the crucial link between gut bacteria and fundamental human biological functions. Because of the varied metabolic genes encoding distinct enzymes and biochemical pathways, the microbiota plays critical roles in energy and nutrition extraction from food (Hou et al., 2022). Furthermore, the creation of bioactive compounds, including vitamins, amino acids, and lipids, is heavily reliant on gut bacteria (Roberfroid et al., 2009). The human microbiota not only protects the host from external pathogens by creating antimicrobial compounds but also plays an essential role in the development of intestinal mucosa and the immune system.

One of the key mechanisms by which the gut microbiota influences health is through the production of short-chain fatty acids (SCFAs), primarily acetate, propionate, and butyrate, which are generated via the fermentation of dietary fibers. SCFAs play a multifaceted role in maintaining gut homeostasis. Butyrate serves as the primary energy source for colonocytes, promoting tight junction integrity and reducing gut permeability, thereby reinforcing the gut barrier. Propionate and acetate, on the other hand, influence systemic metabolism by modulating gluconeogenesis and lipid biosynthesis in the liver (Tan et al., 2014). Beyond metabolic regulation, SCFAs exert anti-inflammatory effects by interacting with G-protein-coupled receptors (GPCRs) and inhibiting histone deacetylases (HDACs), which regulate immune and inflammatory pathways (Yao et al., 2024; Liu et al., 2023).

The immune regulatory role of the microbiota is also mediated through the modulation of nuclear factor kappa B (NF-κB), a critical transcription factor that governs the expression of pro-inflammatory cytokines and antimicrobial peptides. Dysbiosis, or an imbalance in gut microbiota, can lead to overactivation of NF-κB, driving chronic inflammation and increasing susceptibility to inflammatory bowel disease and other gut-related disorders. SCFAs, particularly butyrate, have been shown to inhibit NF-κB activation by preventing the phosphorylation and degradation of inhibitor proteins (IκBs), thereby mitigating inflammatory responses and promoting gut homeostasis (Yao et al., 2024; Liu et al., 2023).

The gut microbiota demonstrates stability, resilience, and symbiotic relationships with the host in healthy environments. Its composition varies significantly across different anatomical sections of the gastrointestinal tract. For example, *Akkermansia muciniphila* resides in the mucus layer of the large intestine and is involved in maintaining intestinal integrity by stimulating mucin production and regulating gut permeability. *Adlercreutzia equolifaciens* is commonly found in the colon, where it metabolizes soy isoflavones into bioactive equol, a compound with antioxidant and estrogenic properties. *Barnesiella* has been discovered in multiple sections of the gastrointestinal tract, including the cecum, colon, and feces, where it contributes to the breakdown of complex carbohydrates and the modulation of immune responses (Afzaal et al., 2022).

Such discrepancies in microbial distribution are primarily attributable to differences in local conditions. The small intestine, for instance, has a rapid transit time and high bile content, while the colon exhibits slower flow rates, a gentler pH, and larger microbial communities dominated by anaerobic species (Afzaal et al., 2022). Aside from regional variation, gut microbiota composition also changes with age. Microbial diversity typically increases from childhood to adulthood and gradually declines after the age of 70, contributing to age-associated vulnerabilities in immune and metabolic health.

## 3 Optimal foods for nurturing gut bacteria

The composition and functionality of gut microbiota are profoundly influenced by specific dietary choices, with certain foods playing a pivotal role in promoting the growth of beneficial bacteria. Microbiota-accessible carbohydrates (MACs), including dietary fibers such as resistant starch, inulin, and pectin, serve as fermentable substrates for gut bacteria, particularly *Bifidobacterium* and *Faecalibacterium prausnitzii*. These fibers stimulate the production of short-chain fatty acids (SCFAs), which are essential for gut homeostasis. For example, resistant starch found in foods like legumes and whole grains has been shown to increase butyrate production, supporting colonic health and reducing gut inflammation. Similarly, inulin, a naturally occurring polysaccharide present in chicory root and Jerusalem artichoke, selectively enhances the abundance of *Bifidobacterium*, contributing to improved gut barrier function and immune modulation (Baxter et al., 2019) (Murga-Garrido et al., 2021).

Polyphenols, bioactive compounds abundant in plant-based foods such as berries, cocoa, and green tea, also play a critical role in supporting gut health. These compounds interact with gut microbiota to produce bioactive metabolites that exert anti-inflammatory and antioxidant effects. For instance, anthocyanins in blueberries have been shown to increase the abundance of *Akkermansia muciniphila*, a bacterium linked to enhanced metabolic health and reduced markers of systemic inflammation (Cano et al., 2024). However, the efficacy of these foods can be significantly influenced by processing methods. High-temperature cooking or refining can degrade fibers and polyphenols, reducing their availability to gut microbes. Minimally processed foods, such as raw fruits, vegetables, and whole grains, retain higher levels of beneficial compounds and are therefore more effective in nurturing gut bacteria (Sejbuk et al., 2024).

Prebiotics, defined as substrates selectively utilized by host microorganisms to confer health benefits, are fundamental to promoting gut health. Common prebiotics include inulin, fructooligosaccharides (FOS), galactooligosaccharides (GOS), and resistant starch, all of which foster the growth of beneficial bacterial taxa such as *Bifidobacterium* and *Faecalibacterium* (Krumbeck et al., 2018). Inulin, naturally present in foods like onions, garlic, and asparagus, is known for its ability to selectively enhance *Bifidobacterium* populations (Davani-Davari et al., 2019). Similarly, FOS, found in bananas, leeks, and artichokes, promotes microbial diversity and supports the production of SCFAs, which are crucial for maintaining gut barrier integrity and reducing inflammation. GOS, commonly derived from dairy products or synthesized for commercial use, has demonstrated particular benefits in infant gut microbiota, simulating the effects of human milk oligosaccharides (Davani-Davari et al., 2019).

Resistant starch, present in foods such as potatoes, green bananas, and legumes, has a unique role in promoting butyrate production. This SCFA is particularly beneficial for colonic health, as it serves as an energy source for colonocytes and reduces intestinal inflammation. However, the efficacy of prebiotics can be influenced by food processing. For example, cooking methods like boiling or steaming generally preserve prebiotic content, whereas high-temperature frying or prolonged storage may degrade these beneficial fibers, reducing their fermentability (Queen and Queen, 2020). To maximize the benefits of prebiotic-rich foods, dietary interventions should prioritize minimally processed options and explore novel methods for enhancing the stability of prebiotics during food preparation (Queen and Queen, 2020).

## 4 Role of microbiota in disease induction

**Figure 2:**
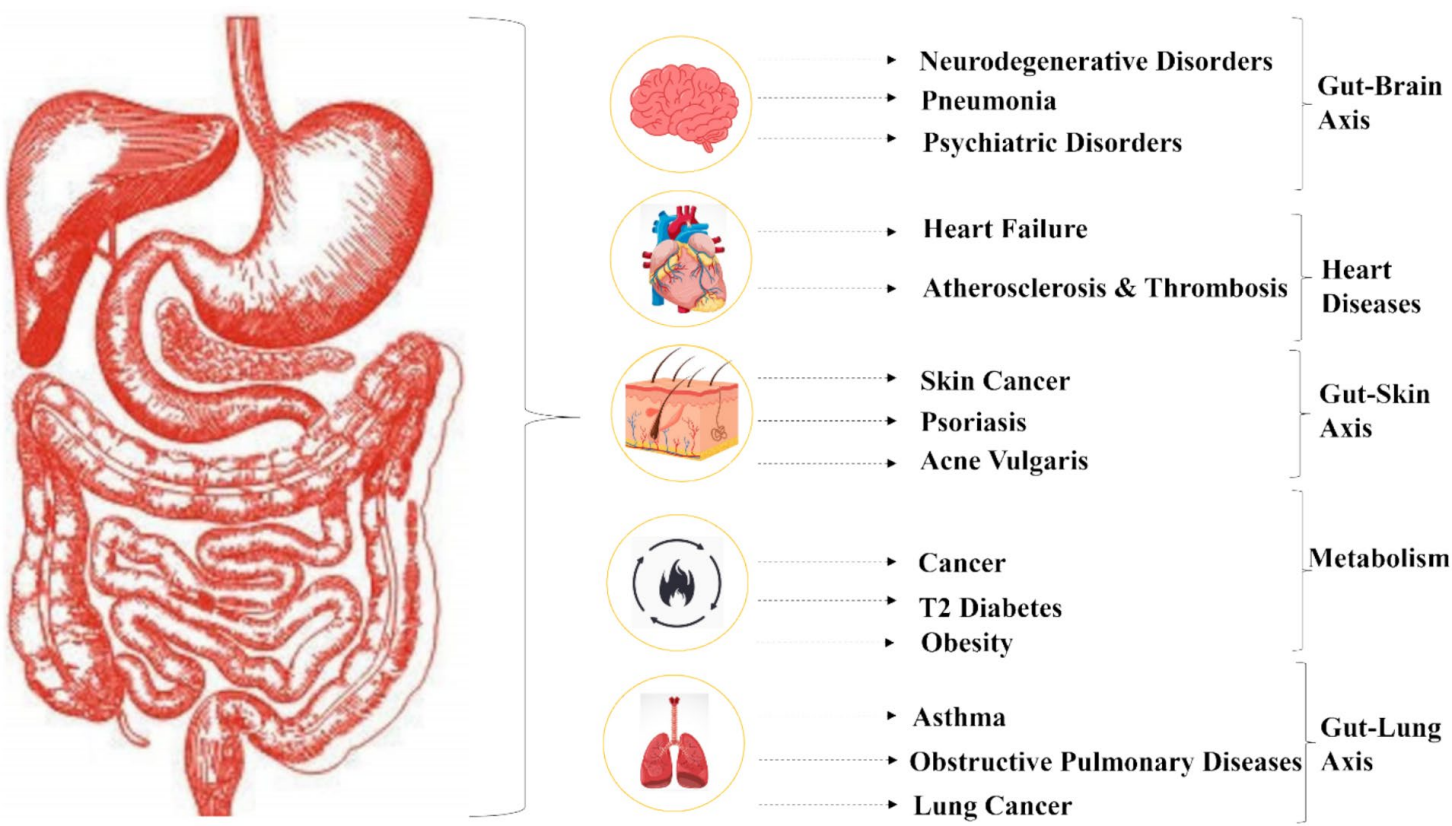
Dysbiosis of the human microbiota.

The intestinal mucosal barrier, which consists of physical, chemical, microbial, and immunological components, serves as one of the body’s major defensive barriers. It protects against bacterial invasion, prevents the entry of foreign antigens and toxins into the circulation, and minimizes water and nutrient loss. This barrier also regulates molecular exchange while supporting the coexistence and colonization of gut bacteria. Disruptions to the gut microbiota’s composition can have significant repercussions, influencing the development of major diseases (Figure 2).

Dysbiosis, characterized by a loss of beneficial microbes and an overgrowth of pathogenic species, plays a central role in disease induction. The intestinal mucosal barrier becomes compromised, leading to increased gut permeability and systemic inflammation. For instance, in inflammatory bowel disease (IBD), the weakened mucus layer of the digestive tract allows luminal bacteria to penetrate epithelial cells (Bull and Plummer 2015), triggering proliferative and inflammatory processes (Parekh et al., 2015). Dysbiosis also affects the production of key microbial metabolites, such as short-chain fatty acids (SCFAs). Reduced levels of butyrate, a critical SCFA, impair gut barrier integrity and exacerbate inflammation, further contributing to the pathogenesis of IBD (Chae et al., 2024).

Emerging evidence has also highlighted the gut’s influence on systemic diseases such as obesity and type 2 diabetes (T2D). Obesity is associated with an increased Firmicutes to Bacteroides/Prevotella ratio, which elevates microbial genes involved in polysaccharide breakdown and raises SCFA production. This enhanced energy extraction from food contributes to weight gain. Additionally, microbial digestion of dietary choline and carnitine, common in Western diets, produces trimethylamine-N-oxide (TMAO), a compound linked to increased cardiovascular disease (CVD) risk. In T2D, dysbiosis results in a proliferation of Proteobacteria, Bacteroidetes, and Firmicutes, disrupting glucose metabolism and contributing to insulin resistance (Muscogiuri et al., 2017).

The gut microbiota’s role extends beyond metabolic and inflammatory disorders to include infectious diseases such as COVID-19. Studies suggest a strong gut-lung axis, wherein the gut microbiota upregulates the synthesis of ACE2, a receptor used by the SARS-CoV-2 virus to enter cells (Wang et al., 2023). Nutraceuticals, including polyphenols and probiotics, have been proposed to support immune responses and potentially mitigate disease severity.

Neurodegenerative diseases, such as Alzheimer’s and Parkinson’s, have also been linked to dysbiosis. Alterations in gut microbiota composition affect the gut-brain axis through microbial metabolites like SCFAs and tryptophan derivatives, influencing neuroinflammation and neurotransmitter synthesis (Junyi et al., 2024). For example, elevated levels of lipopolysaccharides (LPS) in dysbiotic states can disrupt the blood–brain barrier and activate microglia, exacerbating neurodegeneration.

## 5 Gut-brain axis in neurodegenerative diseases

The gut-brain axis represents a dynamic bidirectional communication network linking the gut microbiota and the central nervous system (CNS). This intricate system operates through multiple mechanisms, including microbial metabolites such as short-chain fatty acids (SCFAs), immune signaling, the vagus nerve, and neuroendocrine pathways via the hypothalamic–pituitary–adrenal (HPA) axis. Recent studies underscore the significant role of this axis in influencing brain function, mood regulation, and the pathophysiology of neurodegenerative diseases (Schneider et al., 2024).

Neurodegenerative diseases such as Alzheimer’s disease (AD) and Parkinson’s disease (PD) have been closely linked to gut dysbiosis, a condition characterized by imbalances in microbial composition (Schneider et al., 2024; Kulkarni et al., 2024; Caradonna et al., 2024). In Alzheimer’s disease, gut dysbiosis contributes to increased systemic inflammation through the release of microbial metabolites like lipopolysaccharides (LPS), which compromise the blood–brain barrier integrity. This allows amyloid-beta peptides to accumulate, exacerbating neuroinflammation and neuronal damage (Kulkarni et al., 2024; Caradonna et al., 2024). Similarly, Parkinson’s disease is associated with alterations in gut microbiota composition, particularly a reduction in anti-inflammatory taxa such as *Akkermansia muciniphila*. These changes are thought to facilitate the misfolding and aggregation of *α*-synuclein, a hallmark of PD pathology, and promote its transmission from the enteric nervous system to the CNS via the vagus nerve (Kulkarni et al., 2024; Caradonna et al., 2024).

Microbial metabolites play a pivotal role in modulating neuroinflammatory pathways. SCFAs, including butyrate and propionate, have been shown to regulate microglial activation, which is critical for maintaining CNS homeostasis. A reduction in SCFA levels, often observed in dysbiotic states, is correlated with increased neuroinflammation and cognitive decline (Fodor et al., 2023). Moreover, tryptophan metabolites derived from gut bacteria influence serotonin and kynurenine pathways, both of which are integral to mood regulation and cognitive processes. For instance, studies have highlighted the role of *Bacteroides* species in promoting the synthesis of indole derivatives, which have neuroprotective effects (Fodor et al., 2023).

## 6 Essential gut-friendly bacteria and their byproducts

### 6.1 Akkermansia muciniphila

*Akkermansia muciniphila* is an anaerobic gram-negative anti-obesity bacterium that grows singly or in pairs in mucin-rich medium (Liu et al., 2022). *Akkermansia muciniphila* has garnered significant attention due to its ability to degrade mucus that considers a protective layer that lines the gut and maintains a healthy balance that serves as a barrier against harmful microorganisms as excessive mucus production can lead to a range of health issues, including inflammatory bowel disease (IBD) and obesity (Geerlings et al., 2018; Kim et al., 2021). Mucin layer is primarily made up of gelatinous mucins secreted by goblet cells and also consist of a peptide backbone modified by O-linked glycans. The presence of proline and threonine in the peptide backbone contributes to the unique properties of mucin, including its gel-like consistency and protective function (Pelaseyed et al., 2014). Further, the production of butyrate, a short-chain fatty acid by *Akkermansia muciniphila* not only provides an energy source for the cells lining the colon but also contributes to reducing inflammation and improving gut barrier function (Portincasa et al., 2022). Another metabolite produced by *Akkermansia muciniphila* is propionate, which also has anti-inflammatory effects and regulate glucose metabolism. It also stimulates the production of molecules that promote the growth of beneficial bacteria in the gut, leading to a more diverse and balanced gut microbiota (Rodrigues et al., 2022).

### 6.2 Adlercreutzia equolifaciens

*Adlercreutzia equolifaciens* is a Gram-positive bacterium that thrives in an anaerobic environment, slightly acidic pH range of 6.0 to 6.5 by using culture medium, such as Reinforced Clostridial Medium (RCM). This bacterium is of particular interest due to its ability to produce equol, a metabolite of the soy isoflavone daidzein, which is found in soy products and other legumes. Research has shown that equol has estrogenic and antioxidant properties, which may help alleviate symptoms associated with hormonal imbalances, such as menopause, hot flashes and night sweats (Mayo et al., 2019). Further equol have anti-inflammatory and anti-cancer effects. Individuals who consume a diet rich in soy products are more likely to have a higher abundance of *Adlercreutzia equolifaciens* in their gut (Tuli et al., 2022).

### 6.3 Barnesiella

*Barnesiella* is an obligate anaerobic bacterium that can be cultivated under both anaerobic and microaerophilic conditions at a temperature range of 37–42°C in nutrient-rich media, such as brain-heart infusion (BHI) or tryptic soy broth (TSB), supplemented with appropriate carbon and nitrogen sources (Ubeda et al., 2013)*. Barnesiella* is a commensal bacterium that found reasonably low level in the gastrointestinal tract of humans help in the breakdown of complex carbohydrates, production of SCFAs, modulation of immune responses, facilitate the clearance of intestinal VRE colonization and prevent the spread of highly antibiotic-resistant bacteria (Tao et al., 2021; Ubeda et al., 2013; Aindelis and Chlichlia, 2020).

### 6.4 Christensenella minuta

*Christensenella minuta* is an obligate anaerobe that could be grow optimally under mesophilic conditions, between 30°C to 37°C by using a suitable culture medium, such as a nutrient-rich broth, to support its growth (Kropp et al., 2021; Ang et al., 2023). One of the unique metabolic features of *Christensenella minuta* is its ability to produce butyrate, a short-chain fatty acid linked with various health benefits, including anti-inflammatory and anti-cancer properties. Studies suggest that individuals with higher levels of *Christensenella minuta* may have a lower risk of type 2 diabetes, inflammatory bowel disease and obesity which sparked interest in the use in the development of probiotics and prebiotics (Mazier et al., 2021; Ang et al., 2023). In addition, this bacterium can influence the host’s response to certain medications, such as antidiabetic drugs and suggested that it may contribute to inter-individual differences in drug response and could potentially be targeted for personalized medicine approaches.

### 6.5 Oxalobacter formigenes

*Oxalobacter formigenes* is a unique bacterium that can be grow in anaerobic environment by using Oxalate-Minimal Medium, which consists of oxalate, mineral salts, vitamins and buffer system (Duncan et al., 2002; Daniel et al., 2021). This bacterium resides in the human gastrointestinal tract known for its unique ability to break down and metabolize oxalate, a compound that can form kidney stones in some individuals. Studies have shown that individuals with a higher abundance of *Oxalobacter formigenes* in their gut have a lower risk of developing kidney stones (Chmiel et al., 2022). This bacterium produces an enzyme called oxalyl-CoA decarboxylase, which converts oxalate into a less harmful compound called formate (Karamad et al., 2022).

## 7 Optimal foods for nurturing the growth of gut-friendly bacteria

Research has consistently shown that our dietary choices have a direct impact on the health and resilience of our gut microbiome (Singh et al., 2017; Su and Liu, 2021). The incorporation of prebiotic rich foods, fermented foods, and a wide variety of plants and fruits promote the thriving of these gut microbiome as detailed in Table 1 (Nambiar et al., 2023; Leeuwendaal et al., 2022). The colorful plant foods are not only visually appealing, but they also provide a wide range of health benefits for the microbiome and metabolism. One of the key benefits of colorful foods is their high content of phytochemicals bioactive compounds such as carotenoids, flavonoids, and anthocyanins that have been shown to have numerous health-promoting effects (Samtiya et al., 2021; Khoo et al., 2017).

**Table 1:** Various dietary sources that produce and nourish good gut bacteria.

S. No.	Beneficial bacteria	Substances	Sources	References
1.	*Akkermansia muciniphila*	Polyphenols	Caffeic acid, chlorogenic acid, salvianolic acid A, ferulic, Concord grape polyphenols, puerarin, resveratrol, epigallocatechin gallate, black tea, red wine, grape juice, aronia juice, *Canarium album* extract, arctic berries, flavonoids	Zhou (2017), Visioli et al. (2003), Anhê et al. (2016), Roopchand et al. (2015), Kajla et al. (2015) and Chenbo et al. (2022)
		Alkaloids	Berberine, curcumin caffeine, chlorogenic acid and betaine	
		Capsaicin	Chili peppers	
		Plant-derived carbohydrates	Nonfermentable fiber, wheat dietary fiber, konjac glucomannan, bran, fiber-rich common beans, oligofructose, Inulin-type fructan, stachyose, polysaccharides from spirulina platensis, *Lycium barbarum* polysaccharide and fucoidan	
		Others	Oily fish, walnuts bamboo shoots, rhubarb extract and flaxseed	
2.	*Adlercreutzia equolifaciens*	Isoflavone diet	Tofu, tempeh, and soy milk	Jensen et al. (2021)
3.	*Barnesiella*	Polyphenols	Cherry juices Black raspberry-rich diet *Ganoderma lucidum* mushroom	Ubeda et al. (2013), Daillère et al. (2016), Gu et al. (2019), and Miaoyu et al. (2021)
		Prebiotics		
4.	*Christensenella minuta*	Polyphenols	Red grapes, cranberries, strawberries and blueberries	Mazier et al. (2021), Ang et al. (2023) and Waters and Ley (2019)
5.	*Oxalobacter formigenes*	Prebiotic foods	Prebiotic foods like kimchi, sauerkraut, kefi, spinach, legumes, tea and celery	Kaufman et al. (2008) and Chmiel et al. (2022)

The microbiome is a living dynamic environment where the relative abundance of species may fluctuate daily, weekly, and monthly depending on diet, medication, exercise, and a host of other environmental exposures (Monda et al., 2017; York, 2019).

### 7.1 Carotenoids

Gut microbiota breaks down the carotenoid’s rich foods, into various beneficial gut metabolites such as apocarotenoids and SCFAs having anti-inflammatory, antioxidant, and anticancer properties that contribute to decrease the risk of the development of chronic diseases, cardiovascular diseases, type 2 diabetes, obesity, brain-related diseases and certain types of cancer (Rowles and Erdman, 2020; Rocha et al., 2023; Eroglu et al., 2023; Min et al., 2023). They also act as antioxidants, protecting the body’s cells from damage caused by harmful free radicals. Their ability to protect against lipid peroxidation and damage caused by ROS makes them valuable in maintaining overall health and reducing the risk of chronic diseases (Lobo et al., 2010; Rocha et al., 2023).

Apo-carotenoids exhibit unique characteristics, including higher aqueous solubility and higher electrophilicity, which make them particularly suitable for targeting transcription factors such as NF-κB, PPARγ, and RAR/RXRs (Eroglu et al., 2023). These compounds hold potential for therapeutic applications in the fields of inflammation, metabolic disorders, and cell differentiation. Studies have found that carotenoids can directly influence the composition of the gut microbiota in a positive manner (Rocha et al., 2023). Beta-carotene such as carrots and sweet potatoes can increase the abundance of certain beneficial bacteria, such as *Bifidobacteria* and *Lactobacillus* (Rinninella et al., 2019; Zhiguo et al., 2023; Li et al., 2022a). Lycopene, found in tomatoes, has been shown to increase the levels of bacteria that produce short-chain fatty acids, which are beneficial for gut health (Eroglu et al., 2023). A. Lutein, found in leafy greens, has been associated with a more diverse gut microbiota (Dinsmoor et al., 2019).

### 7.2 Flavonoids

Flavonoid-rich foods including fruits like berries, citrus fruits, vegetables, dark chocolate and tea, can lead to an increase in the diversity and abundance of gut metabolites that break down the flavonoids through fermentation processes into various metabolites compounds that have anti-inflammatory and antioxidant properties and reduce the risk of metabolic disorders such as cardiovascular disease by improving the insulin sensitivity (Joaquim et al., 2023; Wang et al., 2022; Pan et al., 2023). One important group of flavonoids known as flavan-3-ols has been shown to increase the levels of short-chain fatty acids (SCFAs) such as butyrate, which provide energy for the gut epithelial cells and have anti-inflammatory effects (Fotschki et al., 2015). Another bioactive compound flavanols associated with increase the production of urolithins metabolites in the gut that have been shown to have anti-inflammatory, antioxidant, and anticancer properties (Singh et al., 2019). Further flavonoids have also been found to increase the production phenolic acids, benzoic acid derivatives, and microbial-derived compounds that can influence various physiological processes in the body (Rahman et al., 2021). Previous research reported that flavonoids have the ability to modulate the relative abundance by increasing the relative abundance *of Bifidobacterium*, *Lactobacillus*, while decreasing the relative abundance of *Lachnoclostridium* and *Bilophila*, highlights their potential as dietary supplements or functional food ingredients to promote a healthy gut microbiota (Baky et al., 2022; Pan et al., 2023).

### 7.3 Anthocyanins

Anthocyanins that are responsible for bright red, purple, and blue colors to fruits like blueberries, blackberries, purple cabbage and cherries have been shown to have anti-inflammatory and antioxidant effects, as well as potential anti-cancer properties (Khoo et al., 2017; Bahare et al., 2020). They may also help regulate blood sugar levels and improve insulin sensitivity (Fernandes, 2019). One metabolite that has been extensively studied in relation to Anthocyanins increase nitric oxide (NO) metabolite known as signaling molecule that helps in the regulation of blood flow, neurotransmission, and immune responses Geum et al. (2020). Increased levels of nitric oxide relax and dilate blood vessels, leading to improved blood flow and reduced risk of hypertension, heart disease and diabetes (da Silva et al., 2021; Kumar et al., 2022). Studies have consistently shown that anthocyanin leads to an increase in the presence of beneficial bacteria such as *Lactobacillus*, *Bifidobacterium*, *Blautia, Faecalibacterium*, *Prevotella*, *Akkermansia* and stimulate the production SCFAs particularly butyrate (Verediano et al., 2021; Zhong et al., 2023; Liang et al., 2023).

### 7.4 Polyphenols

Polyphenols are a group of compounds that are naturally found in many plant-based foods and beverage that undergo various transformations by the gut bacteria and converted into a wide range of metabolites having antioxidant and anti-inflammatory properties (Gizem et al., 2020; Bertelli et al., 2021). Studies have shown that certain polyphenols, such as those found in green tea, berries, and cocoa, can increase the production of short-chain fatty acids (SCFAs) and branched-chain amino acids (BCAAs) and could be useful in the treatment and prevention of various gastrointestinal disorders (Manach et al., 2004; Kumar Singh et al., 2019; Lippolis et al., 2023). Furthermore, polyphenols have been shown to modulate the composition of the gut microbiota, promoting the growth of beneficial bacteria like *Lactobacillus*, *Lactiplantibacillus* and *Bifidobacterium* while hindering the proliferation of pathogenic strains like *Clostridium* and *Fusobacterium* (Corrêa et al., 2019; Wang et al., 2022; Lippolis et al., 2023; Rahman et al., 2021).

### 7.5 Alkaloids

Alkaloids have been known to possess various biological activities and have been used in traditional medicine for centuries (Heinrich et al., 2021). Studies have shown that certain alkaloids promote the growth of beneficial bacteria that leads to an increase in the production of short-chain fatty acids, such as butyrate, which have numerous health benefits (Feng et al., 2018; Dehau et al., 2023). Furthermore, alkaloids found in coffee, such as caffeine and chlorogenic acid, have been shown to increase the production of certain bile acids in the gut (Iriondo-DeHond et al., 2020; Chen et al., 2023). Bile acids are important for the digestion and absorption of dietary fats and also have regulatory roles in lipid and glucose metabolism (González-Regueiro et al., 2017). Research has shown that berberine has antimicrobial properties, specifically targeting harmful bacteria like *Escherichia coli* (*E. coli*) and *Clostridium difficile* (*C. difficile*), while promoting the growth of beneficial bacteria like Bifidobacterium and *Lactobacillus* (Cheng et al., 2022; Zhang et al., 2021; Peng et al., 2019).

### 7.6 Capsaicin

Research has shown that capsaicin can stimulate the production of certain gut metabolites such as SCFAs known to have various beneficial effects, such as reducing inflammation and improving insulin sensitivity (Song et al., 2017; Kang et al., 2017). Furthermore, capsaicin has been shown to enhance the activity of certain enzymes such as enzyme lipase, which is responsible for breaking down dietary fat (Liu et al., 2021). This increased enzyme activity can lead to a more efficient digestion and utilization of nutrients, ultimately affecting the production of gut metabolites (Menden et al., 2022; Chandra et al., 2020). In addition to its direct effects on gut metabolites, capsaicin has also been found to influence the composition of the gut microbiota and increase the abundance of *Akkermansia muciniphila* (Li et al., 2022b).

## 8 Dietary strategies for optimizing gut microbiota

The composition and functionality of the gut microbiota are profoundly influenced by dietary choices, making nutrition a cornerstone for maintaining microbial health. Specific dietary strategies, including the incorporation of fermented foods, synbiotic combinations, and anti-inflammatory diets, have been shown to promote microbial diversity, enhance the production of beneficial metabolites like short-chain fatty acids (SCFAs), and reduce inflammation. These approaches not only support digestive health but also play a critical role in systemic immunity and metabolic regulation. By tailoring dietary interventions to the unique needs of individuals, it is possible to foster a balanced gut microbiota that contributes to overall well-being and resilience against chronic diseases.

### 8.1 Prebiotic-rich foods

Prebiotics can be found in a variety of foods, including fruits, vegetables, whole grains, and legumes. Some common examples of prebiotic fibers include inulin, fructooligosaccharides (FOS), and galactooligosaccharides (GOS) (Lockyer and Stanner, 2019). The primary benefits of prebiotics are their ability to selectively stimulate the growth of beneficial bacteria, such as *Bifidobacteria, Lactobacilli* and enhance the production of beneficial metabolites like short-chain fatty acids (Markowiak and Śliżewska, 2017; Davani-Davari et al., 2019). Prebiotic rich foods such as Chicory root and Jerusalem Artichokes are excellent sources of inulin, a fiber that serves as a fuel for the beneficial gut bacteria (Carlson et al., 2018). Similarly, the content of pectin in apples and resistant starch in bananas acts as nourishment for good bacteria (Englyst and Cummings, 1986; Leonel and Alvarez-Leite, 2012).

Other prebiotic foods like oats, barley, and quinoa promote healthy gut bacteria by providing good source of prebiotic fibers (Slavin, 2013). Legumes foods, including lentils, chickpeas, and beans, are known for their high nutritional value and are an excellent source of prebiotics that promote the growth of beneficial bacteria in the gut (Kadyan et al., 2022). The content of fructooligosaccharides (FOS) in garlic and onions, and inulin and oligofructose in asparagus vegetables act as prebiotics by stimulating the growth of beneficial gut bacteria (Zhang et al., 2013; Guillamón et al., 2021).

### 8.2 Fermented foods

Fermented foods are a rich source of probiotics that enhance gut health by introducing beneficial bacteria and improving microbial diversity. Popular examples include yogurt, kimchi, and sauerkraut. These foods support gut integrity by increasing levels of *Lactobacillus* and *Bifidobacterium*, which produce metabolites like short-chain fatty acids (SCFAs) that reduce inflammation and strengthen the gut barrier (Dimidi et al., 2019). In addition to these widely studied examples, global fermented foods such as miso and natto from Japanese cuisine, kefir from Eastern Europe, and dosa from India provide unique probiotic strains and bioactive compounds. Miso and natto, for instance, contain *Bacillus subtilis*, which has been shown to promote immune regulation and reduce markers of systemic inflammation (Oshiro et al., 2021). Kefir is particularly rich in lactic acid bacteria and yeast, offering a broad spectrum of probiotics with antimicrobial and gut-stabilizing properties (Tingirikari et al., 2024).

Fermented foods provide a natural and delicious way to support a healthy gut microbiome. With their probiotic properties, they offer numerous benefits, including improved digestive health, enhanced immune function, increased nutrient availability, and potential mental well-being (Leeuwendaal et al., 2022). Yogurt is a widely consumed fermented dairy product that augments beneficial bacteria, such as *Lactobacillus* and *Bifidobacterium* (Lisko et al., 2017). Kimchi is mainly produced by fermented vegetables, including cabbage, radishes, and garlic that contain *Lactobacillus* bacteria, which are known to promote gut health and improve digestion (Dimidi et al., 2019).

Another fermented food known as Kombucha made from a fermented tea beverage that is rich in bioactive compounds and a variety of beneficial acetic acid bacteria and yeasts, contributing to a healthy gut microbiome (Kitwetcharoen et al., 2023). A previous study conducted on rats revealed that tempeh, a fermented soybean product, has the potential to enhance the production of immunoglobulin A (IgA) and modulate the composition of gut microbiota. In addition to the study on rats, research involving supplementation of tempeh in humans for 16 days led to a significant increase in the abundance of beneficial gut bacteria like *Akkermansia muciniphila* (Stephanie et al., 2017).

Practical dietary recommendations include incorporating a variety of fermented foods into daily meals. For example, pairing kimchi with rice or adding yogurt to smoothies can provide both flavor and health benefits. Future research should explore the synergistic interactions between probiotics in fermented foods and the resident gut microbiota to optimize therapeutic applications.

### 8.3 Synbiotic foods

Synbiotic foods provide a convenient and effective way to support a healthy gut microbiome (Jiang et al., 2022). By combining prebiotics and probiotics, these foods offer numerous health benefits, including improved digestion, enhanced immune function, increased nutrient absorption, and reduced inflammation (Yadav et al., 2022). Incorporating synbiotic foods into a balanced diet can be a valuable strategy for promoting overall health and well-being (Pandey et al., 2015). Synbiotic food having a combination of arabinose, lactulose and *Lactobacillus plantarum*, have gained attention for their potential to effectively regulate blood glucose, blood lipid, and body weight in patients with Type 2 Diabetes Mellitus (T2DM) (Jiang et al., 2022). Another preparation known as a banana smoothie made with kefir or yogurt is a delicious and nutritious beverage that maximizes the benefits for gut bacteria. Stir-fry made with tempeh, asparagus, garlic, and leeks prompts, you can actively healthy gut microbiome. The combination of yogurt and fruits such as blueberries creates a synbiotic effect, where the probiotics from yogurt and the prebiotics from blueberries work together to promote the growth and activity of healthy gut bacteria (Fernandez and Marette, 2017).

### 8.4 Anti-inflammatory foods

Anti-inflammatory foods such as fatty fish, fruits, vegetables, whole grains, and spices have long been recognized for their potential health benefits by supporting a diverse and balanced gut microbiome, reducing inflammation and promoting overall gut health (Bagheri et al., 2022). Fish like salmon, sardines, and anchovies in diet can have a positive impact on gut health. Their omega-3 fatty acids and ability to increase healthy gut bacteria make them a valuable addition to an anti-inflammatory diet (Costantini et al., 2017). Flax seeds are rich in dietary fiber, including both soluble and insoluble fiber that act as prebiotics and provide nourishment for the beneficial bacteria in the gut (Kajla et al., 2015; Mueed et al., 2022). When the gut bacteria ferment the fiber from flax seeds, they produce short-chain fatty acids (SCFAs), such as butyrate that help in maintaining a healthy gut environment (Arora et al., 2019).

## 9 Impact of diet on microbial ecology in the gut

Diet plays a pivotal role in shaping the composition, diversity, and metabolic activity of the gut microbiota. Long-term dietary patterns, including plant-based and high-protein diets, create distinct microbial environments that influence host health. Microbiota-accessible carbohydrates (MACs), a subset of dietary fibers, serve as the primary energy source for gut bacteria, fostering the growth of beneficial taxa and stimulating the production of short-chain fatty acids (SCFAs) (De Filippo et al., 2010). MACs are composed of complex polysaccharides, such as resistant starches, inulin, and pectin, that are indigestible by host enzymes but fermentable by gut microbes. These carbohydrates selectively enhance the abundance of health-promoting bacteria like *Bifidobacterium* and *Faecalibacterium prausnitzii*, both of which are associated with anti-inflammatory effects and improved gut barrier integrity (Hills et al., 2019). For example, resistant starch found in legumes and whole grains increases butyrate production, which supports colonic health. Similarly, inulin from chicory root and Jerusalem artichokes promotes the proliferation of *Bifidobacterium*, contributing to gut homeostasis and reduced systemic inflammation. These carbohydrates are made up of monosaccharides linked together by various forms of glycosidic connections. Any major differences in their chemical makeup, solubility, and size classify these carbohydrates into a wide range of biological niches. Dietary fibers are crucial sources of energy for the bacteria that live in the colon and cecum. In addition to reducing microbial diversity and short chain fatty acid (SCFA) production, a low intake of dietary fiber causes the gut microbial metabolism to shift toward the use of less advantageous substrates. The mucus barrier is harmed by a protracted absence of dietary fibers, which is also linked to an increase in the number of bacteria that break down mucins, like *Akkermansia muciniphila* (Makki et al., 2018).

**Figure 3:**
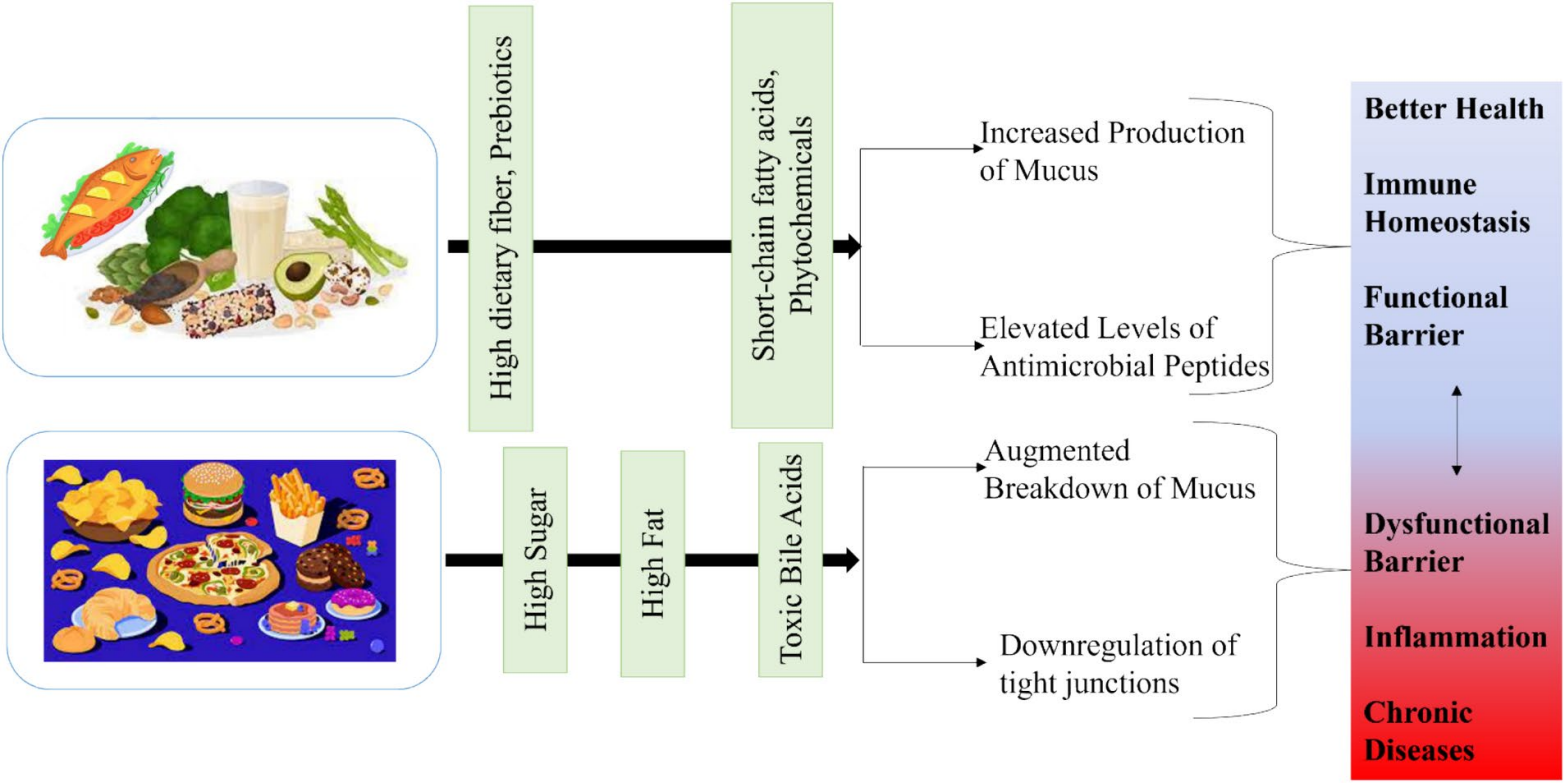
Effect of high and low fiber diet on gut microbiota.

As a result, a lack of dietary fibre and an increase in sugar and fat in human diets may lead to the extinction of particular bacterial species. These changes may lead to dysfunctions, which could exacerbate existing conditions like IBD, allergies, colorectal cancer, autoimmune illnesses, obesity, etc. A diet high in fiber helps to maintain a healthy gut microbiota that is more diverse and performs activities like producing short-chain fatty acids (SCFAs) (Figure 3). Low fiber intake, a diet high in protein and sugar, and an industrialized diet all contribute to altered gut bacterial function, including a significant decrease in their capacity to produce SCFAs, which is linked to the emergence of chronic inflammatory diseases (Makki et al., 2018).

## 10 Microbiome-targeted therapies

Advancements in microbiome research have led to the development of innovative therapies aimed at modulating gut microbiota to improve health outcomes, including CNS-related disorders. Microbiome-targeted therapies encompass probiotics, prebiotics, postbiotics, dietary interventions, and fecal microbiota transplantation (FMT), each offering unique mechanisms to restore microbial balance and enhance host health (Loh et al., 2024).

Probiotics, defined as live microorganisms that confer health benefits when consumed in adequate amounts, have garnered attention for their potential to influence the gut-brain axis. Specific strains, such as *Lactobacillus rhamnosus* and *Bifidobacterium longum*, have shown promise in alleviating symptoms of anxiety and depression by modulating the HPA axis and reducing systemic inflammation (Schächtle and Rosshart, 2021). Moreover, these probiotics enhance the production of neuroactive compounds like gamma-aminobutyric acid (GABA), which exerts calming effects on the CNS (Schächtle and Rosshart, 2021; Loh et al., 2024).

Prebiotics, including dietary fibers and oligosaccharides, serve as substrates for beneficial gut bacteria, stimulating the production of SCFAs and other bioactive metabolites. For example, inulin and fructooligosaccharides have been shown to increase butyrate levels, which play a critical role in reducing neuroinflammation and preserving neuronal integrity (Anand et al., 2025). Prebiotic interventions also hold potential for personalized nutrition, wherein diets are tailored to enhance specific microbial taxa associated with improved CNS health.

Fecal microbiota transplantation (FMT), a procedure involving the transfer of stool from a healthy donor to a recipient, has emerged as a promising therapeutic avenue for neuropsychiatric and neurodegenerative disorders. Preliminary clinical trials suggest that FMT can restore microbial diversity and ameliorate symptoms in conditions such as autism spectrum disorder and depression (Zhang et al., 2023; Peery et al., 2024). However, the standardization of FMT protocols and the identification of specific microbial communities responsible for therapeutic effects remain critical challenges (Zhang et al., 2023).

Future research should focus on the development of “designer probiotics” tailored to target specific diseases and the integration of microbiome data with genomic and metabolomic profiles to advance precision medicine. Additionally, leveraging computational tools to model host-microbiota interactions could facilitate the identification of novel therapeutic targets, paving the way for next-generation microbiome-based treatments.

## 11 Conclusion

The study of the human gut microbiota has advanced significantly in recent years, revealing its profound influence on the host’s metabolism, physiology, and immune system. Numerous factors, including nutrition, host genetics, age, medications, and lifestyle, shape the composition and functionality of the gut microbiota. These dynamic interactions play a pivotal role in human health, and alterations in gut microbial composition are now recognized as key contributors to the development of various diseases. A deeper understanding of the interplay between diet and microbiota holds the potential to develop tailored nutritional strategies aimed at reducing the prevalence of chronic inflammatory disorders.

Emerging evidence highlights the critical role of plant-based foods rich in phytochemicals and other specific dietary components in promoting the growth and maintenance of beneficial gut bacteria. However, challenges such as absorption, bioavailability, dietary interactions, and individual variations in metabolism can limit the efficacy of phytochemicals in fostering a healthy gut microbial community. The highly dynamic nature of gut microbiota, influenced by environmental factors, further complicates these interventions. Addressing issues such as standardization of dietary recommendations, variability in individual responses, and the long-term maintenance of dietary changes is essential for optimizing the benefits of specific foods on gut health.

In this review, we explored the intricate roles of beneficial gut bacteria and the significant impact of optimal diets on human health. Our findings underscore the transformative potential of personalized nutrition, customized to individual microbiota profiles, in revolutionizing healthcare. By leveraging the symbiotic relationship between gut bacteria and dietary patterns, targeted dietary interventions and preventive strategies can be developed to address chronic diseases. However, implementing such personalized recommendations on a large scale requires a paradigm shift in healthcare systems, moving towards individualized care. Ethical concerns, particularly related to privacy and the use of data in precision medicine, also present formidable challenges that must be carefully navigated.

Advancements in microbiome-targeted therapies, including probiotics, prebiotics, and synbiotics, along with the integration of artificial intelligence (AI), offer promising avenues for tailoring dietary interventions. AI-driven models can integrate microbiome, metabolome, and genomic data to generate precise nutritional recommendations, optimizing dietary strategies at an individual level. Future research should prioritize understanding the mechanisms underlying host-microbiota interactions, improving the bioavailability of key dietary components, and evaluating the long-term effects of dietary interventions on microbial ecology. Combining these scientific insights with robust ethical frameworks is essential to fully harness the potential of gut microbiota in promoting health and preventing disease. Through a multidisciplinary approach, we can unlock innovative solutions that bridge scientific discovery and practical implementation, advancing healthcare and improving lives.
